# Prevalence of sexually transmitted infections among incarcerated men in Prison Complex: a cross-sectional study, Santa Catarina, 2023-2024

**DOI:** 10.1590/S2237-96222026v35e20250140.en

**Published:** 2026-02-16

**Authors:** Aline Ferreira Farias, Maria Laura Gava, Symonara Karina Medeiros Faustino Vieira, Gabriela Sousa De Araujo, Cassandra Loureiro Mangabeira, Eliane Traebert, Jefferson Traebert, Daisson José Trevisol, Fabiana Schuelter-Trevisol

**Affiliations:** 1 Universidade do Sul de Santa Catarina, Programa de Pós-Graduação em Ciências da Saúde, Tubarão, SC, Brazil

**Keywords:** HIV, Syphilis, Hepatitis B, Hepatitis C, Prisoners., VIH, Sífilis, Hepatitis B, Hepatitis C, Prisioneros.

## Abstract

**Objective:**

To estimate the prevalence and associated factors of HIV, viral hepatitis B and C, and syphilis among incarcerated men in the Prison Complex of Santa Catarina.

**Methods:**

This cross-sectional study was conducted among incarcerated men in three prison units in Santa Catarina from August 2023 to January 2024. Questionnaires were administered, and participants’ health records were reviewed based on simple random sampling. Crude and adjusted prevalence ratios, along with their corresponding confidence intervals, were calculated for the outcomes and independent variables.

**Results:**

A total of 555 men were included in the study. The mean age was 31.8±9.5 years. The predominant sociodemographic profile was of young, White, low-educated, single individuals. In the sample, risk behaviors such as having tattoos (87.2%), using inhaled drugs (55.5%), and low adherence to condom use (15.2%) were identified. The overall prevalence of tested sexually transmitted infections (STI) was 16.8%, and 32.6% self-reported current or past STI. The prevalence of HIV was 3.4%; syphilis, 12.4%; hepatitis B, 1.3%; and hepatitis C, 1.8%. Syphilis was associated with a higher number of sexual partners, while hepatitis B and C were associated with older age.

**Conclusion:**

STI prevalence rates were higher than in the general population, based on diagnoses at prison entry. There were shortcomings in treatment compliance follow-up and retesting, which are essential to interrupt transmission chains.

## Resumen

**Objetivo:**

Estimar la prevalencia y los factores asociados al VIH, las hepatitis virales B y C y la sífilis en hombres privados de libertad del Complejo Penitenciario de Santa Catarina.

**Métodos:**

Se trata de un estudio transversal con hombres privados de libertad en tres centros penitenciarios de Santa Catarina, realizado entre agosto de 2023 y enero de 2024. Se aplicaron cuestionarios y se revisaron las fichas sanitarias de los participantes, a partir de una muestra aleatoria simple. Se calcularon las razones de prevalencia brutas y ajustadas y los intervalos de confianza entre los resultados y las variables independientes.

**Resultados:**

Se estudió a 555 hombres. La edad media fue de 31,8 ± 9,5 años. El perfil sociodemográfico predominante era de jóvenes, blancos, con bajo nivel educativo y solteros. En la muestra se identificaron comportamientos de riesgo como tatuajes (87,2 %), consumo de drogas inhaladas (55,5 %) y baja adherencia al uso del preservativo (15,2 %). La prevalencia general de las infecciones de transmisión sexual (ITS) analizadas fue del 16,8 %, y el 32,6 % autoinformaron ITS actuales o previas. La prevalencia del VIH fue del 3,4 %; la sífilis, del 12,4 %; la hepatitis B, del 1,3 %; y la hepatitis C, del 1,8 %. La sífilis se asoció con un mayor número de parejas sexuales, y la hepatitis B y C, a una edad más avanzada.

**Conclusión:**

Las tasas de prevalencia de ITS fueron más altas que en la población general a partir del diagnóstico al ingresar al sistema penitenciario. Hubo fallas en el seguimiento de la adherencia al tratamiento y en la repetición de las pruebas para interrumpir la cadena de transmisión.

**Ethical aspects**

This research respected ethical principles, having obtained the following approval data:

Research ethics committee: Universidade do Sul de Santa Catarina

Opinion number: 5,591,802

Approval date: 19/8/2022

Certificate of submission for ethical appraisal: 59773922.2.0000.5369

Research ethics committee: Universidade do Sul de Santa Catarina

Opinion number: 6,087,745

Approval date: 29/5/2023

Certificate of submission for ethical appraisal: 68717023.8.0000.0261

Informed consent record: Obtained from all participants prior to data collection

## Introduction

Brazil has the third largest prison population in the world ^1^. The context of incarceration in the country is marked by adverse conditions, such as overcrowding, structural precariousness, poor hygiene, violence, shortcomings in health care, and unequal power relations ^2^ ^,^ ^3^. These factors contribute to a highly vulnerable environment, favoring risk behaviors that increase the transmission of infectious agents. In this scenario, sexually transmitted infections (STI), such as human immunodeficiency virus (HIV), syphilis, hepatitis B, and hepatitis C, are significantly more prevalent among incarcerated individuals than in the general population ^4^ ^,^ ^5^. 

These infections are associated with behaviors such as unprotected sexual intercourse, drug use, and sharing of sharp objects or personal hygiene items ^4^ ^,^ ^6^. The high rate of STI in this population represents a significant public health challenge, especially considering that incarcerated individuals are part of key populations - those most exposed to infection and with limited access to prevention and treatment ^7^ ^,^ ^8^. In 2017, 3.8% of incarcerated individuals worldwide were living with HIV and 15.1% with hepatitis C ^7^.

In the prison system of Dallas, Texas, STI rates in correctional facilities remained alarming, reinforcing the need for surveillance and effective public policies ^4^. Beyond individual impacts, high infection rates represent a collective risk both inside and outside prisons. In Rio de Janeiro prisons, deaths from infectious diseases were five times more frequent than among the general population, with HIV/AIDS accounting for 43.0% of these deaths ^9^.

Prisons are highly complex environments, where factors such as unfavorable socioeconomic conditions, limited access to health services, and risk behaviors - such as syringe sharing and unprotected sex between men - contribute to the spread of STI ^8^ ^,^ ^10^.

This study aimed to estimate the prevalence and associated factors of HIV, hepatitis B and C, and syphilis among incarcerated men in the Prison Complex of Santa Catarina.

## Methods

Study Design

This was a cross-sectional study. 

Setting

The study focused on male prisoners at the Penitentiary and the Male Prison in Florianópolis, as well as the Male Penitentiary in Tubarão, Santa Catarina, between August 2023 and January 2024. At the time of data collection, the Florianópolis Penitentiary had an official capacity of 1,384 inmates (but housed 1,561 inmates); the Florianópolis Male Prison had an official capacity of 475; and the Tubarão Penitentiary, 370. The number of individuals per cell varied, but on average, there were six to eight incarcerated men per cell.

Participants

The study included all incarcerated men who underwent rapid testing for HIV, syphilis, and hepatitis B and C and signed an informed consent form after being individually invited to participate, thereby ensuring their autonomy. 

Individuals who did not provide their name or inmate number, which prevented data collection related to rapid and confirmatory tests and health conditions, were excluded. Those hospitalized in the Custody and Psychiatric Treatment Hospital of the Florianópolis Penitentiary were also excluded.

Variables

The study included sociodemographic data, health history, and risk behaviors, as detailed below. 

Sociodemographic data: Age categorized into ranges (18-29, 30-39, 40-49, 50-59, ≥60 years); race/skin color (White, Black/Brow, Indigenous, Asian); educational level (incomplete and complete elementary school, incomplete and complete high school, incomplete and complete higher education); marital status (single, married/in a common-law marriage, separated/divorced, widowed). 

Health history and risk behaviors: History of surgery (yes, no), previous blood transfusion (yes, no), having tattoos (yes, no) or piercings (yes, no), use of inhaled (yes, no) and injectable (yes, no) illicit drugs, sharing of objects such as razor blades (yes, no), whether had or has sex in prison (yes, no), sexual orientation (heterosexual, homosexual, bisexual), number of sexual partners in life (dichotomized at the median: 0-34, >34) and in the past 12 months (0-1, >1), condom use (always, most of the time, occasionally, never), vaccination against hepatitis B (yes, no), and self-reported diagnosis of previous or current STI (yes, no). 

Dependent variables: HIV infection (reactive, non-reactive), syphilis (reactive, non-reactive), hepatitis B (reactive, non-reactive), and hepatitis C (reactive, non-reactive).

Data sources and measurement

The variables were collected using a self-administered questionnaire developed by the researchers, with closed and multiple-choice questions. Additional information was obtained from the prison health units’ medical records and transcribed into the database. Results of rapid and confirmatory tests for HIV, syphilis, and hepatitis B and C performed at prison entry were reviewed. Regarding information obtained directly from participants and without the possibility of data verification, if the information was unknown or the respondent refused to answer, the response category was standardized as unknown data.

Bias

The data collection instrument was not validated. Although probabilistic sampling was used, some refusals and restrictions due to the high risk associated with the participants may have influenced the results.

Study Size

The sample size was calculated using the OpenEpi program, considering a total population of 2,260 individuals, an unknown outcome prevalence (P=50%), and a 95% confidence interval, resulting in a minimum sample size of 329 participants. An additional 30.0% was added as a safety margin for potential losses. Simple random sampling was conducted based on the numbering of prison blocks, and data collection feasibility was considered, as access was not permitted in some areas due to security reasons or the absence of a suitable location for administering questionnaires.

Data collection

Selected participants were invited to participate in the study. Upon agreement and signature of the informed consent form, they completed a self-administered questionnaire in the presence of a researcher, who was available to clarify any doubts regarding the instrument.

The questionnaire included the collection of sociodemographic data related to incarceration, sexual life, and risk behaviors. The questions were based on instruments previously used in studies on this topic, according to a literature review. Classrooms in authorized sectors were used for data collection. Small groups of participants were selected through cell-based draws until the required sample size was reached. Subsequently, the medical records found in the Santa Catarina Integrated Public Security System were reviewed to verify the results of HIV, syphilis, and hepatitis B and C serological tests, as well as complementary clinical variables. 

The Santa Catarina State Health Department provided the rapid tests used, which showed high sensitivity and specificity. As the tests were administered upon entry into the prison system, and some participants had been incarcerated for several years, the test marks may have varied; however, this was not specified in the records. 

The rapid tests consisted of immunochromatographic assays for detecting anti-HIV antibodies, syphilis IgG and IgM antibodies, anti-hepatitis C antibodies, and HBsAg. All reactive results were forwarded to the municipality’s specialized service for confirmatory testing in accordance with the Brazilian Ministry of Health’s protocol for case notification. Syphilis was confirmed using the Venereal Disease Research Laboratory test. For positive HIV tests, a second serological test was performed and confirmed by viral load via polymerase chain reaction (PCR), which was also used for hepatitis B and C detection. Although the medical records showed confirmed infection, the confirmatory tests were not always filed at the Prison Complex. 

Control retesting of syphilis serological markers should be performed every three months to monitor treatment and detect reinfection. It was noted that this procedure was not always followed, nor was the retesting of non-reactive cases during their stay at the institution. For this reason, the positive result upon entry into the prison system was considered in this study. 

Statistical methods

The collected information was organized in a Microsoft Excel 2016 database. Statistical analysis was performed using SPSS software v. 21 (IBM, Armonk, NY, United States). The Kolmogorov-Smirnov test was used to assess the normality of quantitative data. 

Quantitative variables were described using measures of central tendency and data dispersion. Continuous variables, such as age and the number of partners in life and in the last 12 months, were dichotomized at the median of their respective distributions. Qualitative variables were described using absolute and relative frequencies. Differences in proportions were tested using Pearson’s chi-square test, while differences in means were tested using Student’s t-test or nonparametric equivalents, depending on the suitability of the data. The statistical significance level adopted was 5%, with a 95% confidence interval.

Statistical analysis was conducted using the Statistical Package for the Social Sciences (SPSS), version 18.0 (SPSS Inc., Chicago, IL, United States). Quantitative variables were described using measures of central tendency and dispersion. Crude and adjusted prevalence ratios (PR) and their respective 95% confidence intervals (95%CI) were estimated using Poisson regression with robust variance. To adjust for confounding variables in the three models studied, variables with p-value<0.20 in the bivariate analysis were included. The significance level adopted was 5%.

## Results

A total of 555 incarcerated men were studied between August 2023 and January 2024. The participants’ mean age was 31.8 years (standard deviation ± 9.5), with a range of 18 to 76 years (Table 1). Data related to risk factors and vulnerability associated with STI were observed based on self-reported information provided by participants in the study (Table 2). Data on behavioral risk factors related to sexual behavior, self-reported by study participants, were presented, along with statements regarding current or previous STI infections and hepatitis B vaccination (Table 3).

According to rapid tests performed upon entry into the prison system, 93 individuals (16.8%) had reactive results for at least one of the four infections tested. The highest prevalence was syphilis (69; 12.4%), followed by HIV (19; 3.4%), hepatitis C (10; 1.8%) and hepatitis B (7; 1.3%). There were ten cases of coinfection: three cases of HIV/syphilis, two of HIV/hepatitis B, two of HIV/hepatitis B/hepatitis C, two of syphilis/hepatitis C, and one of syphilis/hepatitis B. 

The results of the bivariate analysis between independent variables and the four study outcomes were presented (Table 4). Older age and non-adherence to condom use were associated with HIV infection. For hepatitis B, older age and injection drug use were also associated with infection. In the case of syphilis and hepatitis C, no independent variable was associated with the outcome. However, in the crude and adjusted analyses of independent variables and outcomes, only older age remained independently associated with hepatitis B infection (Table 5).

**Table 1:** Sociodemographic profile of incarcerated men. Male Prison Complex in Tubarão and Florianópolis, 2022-2023 (n=555)

Variable	n (%)
Age group (years)	
18-29	272 (49.0)
30-39	178 (32.1)
40-49	75 (13.5)
50-59	22 (4.0)
≥60	8 (1.4)
Race/skin color	
White	277 (49.9)
Black/Brown	268 (48.3)
Indigenous	7 (1.3)
Asian	3 (0.5)
Education level	
Incomplete elementary school	285 (51.4)
Complete elementary school	74 (13.3)
Incomplete high school	116 (20.9)
Complete high school	48 (8.6)
Incomplete higher education	22 (4.0)
Complete higher education	10 (1.8)
Marital Status	
Single	303 (54.6)
Married/In a common-law marriage	213 (38.4)
Separated/Divorced	33 (5.9)
Widowed	6 (1.1)

**Table 2:** Risk factors and vulnerability associated with sexually transmitted infections among incarcerated men. Male Prison Complex in Tubarão and Florianópolis, 2023-2024 (n=555)

Variable	n (%)
Previous surgery	
Yes	270 (48.7)
No	271 (48.8)
Unknown	14 (2.5)
Previous blood transfusion	
Yes	159 (28.7)
No	367 (66.1)
Ignored	29 (5.2)
Having tattoos	
Yes	490 (88.2)
No	63 (11.4)
Ignored	2 (0.4)
Having piercings	
Yes	44 (8.0)
No	503 (90.6)
Unknown	8 (1.4)
User of injection illicit drug	
Yes	48 (8.6)
No	466 (84.0)
Unknown	41 (7.4)
User of inhaled illicit drugs	
Yes	308 (55.5)
No	218 (39.3)
Unknown	29 (5.2)
Sharing of personal hygiene objects	
Yes	107 (19.3)
No	407 (73.3)
Unknown	41 (7.4)

**Table 3:** Data related to sexual issues and behaviors, including treatment and prevention of sexually transmitted infections among incarcerated men. Male Prison Complex in Tubarão and Florianópolis, 2023-2024 (n=555)

Variable	n (%)
Sexual orientation	
Heterosexual	530 (95.5)
Homosexual	5 (0.9)
Bisexual	20 (3.6)
Had or has sex in prison	
Yes	143 (25.8)
No	401 (72.3)
Unknown	11 (1.9)
Number of sexual partners in life	
0-34	128 (23.1)
>34	125 (22.5)
Unknown	302 (54.4)
Number of sexual partners in the past 12 months	
0-1	267 (48.1)
>1	95 (17.1)
Unknown	193 (34.8)
Has or had any sexually transmitted infections	
Yes	178 (32.6)
No	352 (64.5)
Unknown	25 (2.9)
Condom use	
Always	83 (15.0)
Most of the time	114 (20.5)
Occasionally	188 (33.8)
Never	143 (25.8)
Unknown	27 (4.9)
Vaccination against hepatitis B	
Yes	122 (22.0)
No	135 (24.3)
Unknown	298 (53.7)

**Table 4:** Association between HIV, syphilis, hepatitis B and C, and variables of interest. Male Prison Complex in Florianópolis and Tubarão, 2023-2024 (n=555)

	HIV			Syphilis			Hepatitis B			Hepatitis C		
	Reactive	Non-reactive	p-value	Reactive	Non-reactive	p-value	Reactive	Non-reactive	p-value	Reactive	Non-reactive	p-value
	n (%)	n (%)		n (%)	n (%)		n (%)	n (%)		n (%)	n (%)	
Age group (years)			0.042			0.307			0.008			0.052
18-29	5 (1.9)	261 (98.1)		38 (14.6)	222 (85.4)		-	268 (100.0)		2 (0.8)	250 (99.2)	
>29	14 (5.1)	260 (94.9)		31 (11.6)	236 (88.4)		7 (2.6)	267 (97.4)		8 (3.2)	243 (96.8)	
Race/skin color			0.092			0.182			0.488			0.354
White	13 (4.9)	254 (95.1)		29 (11.1)	232 (88.9)		4 (1.5)	264 (98.5)		6 (2.4)	241 (97.6)	
Non-white	6 (2.2)	267 (97.8)		40 (15.0)	226 (85.0)		3 (1.1)	271 (98.9)		4 (1.6)	252 (98.4)	
Education level (years)			0.374			0.402			0.228			0.230
0-8	14 (4.0)	332 (96.0)		48 (14.0)	295 (86.0)		6 (1.7)	345 (98.3)		8 (2.5)	312 (97.5)	
>8	5 (2.6)	189 (97.4)		21 (11.4)	163 (88.6)		1 (0.5)	190 (99.5)		2 (1.1)	181 (98.9)	
Previous surgery			0.111			0.449			0.062			0.196
Yes	13 (4.9)	253 (95.1)		36 (14.0)	222 (86.0)		6 (2.3)	259 (97.7)		7 (2.8)	246 (97.2)	
No	6 (2.3)	255 (97.7)		30 (11.7)	226 (88.3)		1 (0.4)	263 (99.6)		3 (1.3)	235 (98.7)	
Blood transfusion			0.633			0.073			0.350			0.457
Yes	6 (3.9)	148 (96.1)		13 (8.7)	137 (91.3)		3 (1.9)	151 (98.1)		2 (1.4)	142 (98.6)	
No	11 (3.1)	347 (96.9)		51 (14.5)	301 (85.5)		4 (1.1)	357 (98.9)		7 (2.1)	326 (97.9)	
Tattoo			0.623			0.271			0.443			0.683
Yes	17 (3.6)	460 (96.4)		58 (12.4)	411 (87.6)		7 (1.5)	475 (98.5)		9 (2.0)	436 (98.0)	
No	2 (3.2)	60 (96.8)		10 (17.5)	47 (82.5)		-	59 (100.0)		1 (1.8)	56 (98.2)	
Piercing			0.550			0.863			0.546			0.542
Yes	1 (2.4)	41 (97.6)		6 (14.0)	37 (86.0)		-	44 (100.0)		1 (2.7)	36 (97.3)	
No	18 (3.7)	474 (96.3)		62 (13.0)	414 (87.0)		7 (1.4)	483 (98.6)		9 (2.0)	451 (98.0)	
Object Sharing			0.313			0.078			0.715			0.394
Yes	2 (1.9)	103 (98.1)		19 (17.9)	87 (82.1)		1 (0.9)	105 (99.1)		1 (1.0)	99 (99.0)	
No	14 (3.5)	382 (96.5)		44 (11.5)	340 (88.5)		4 (1.0)	392 (99.0)		8 (2.2)	360 (97.8)	
Injection illicit drug use			0.487			0.547			0.013			0.577
Yes	2 (4.3)	45 (95.7)		7 (15.6)	38 (84.4)		3 (6.3))	45 (93.8)		1 (2.4)	41 (97.6)	
No	15 (3.3)	438 (96.7)		55 (12.4)	388 (87.6)		3 (0.7)	451 (99.3)		8 (1.9)	415 (98.1)	
Inhaled illicit drug use			0.193			0.081			0.665			0.362
Yes	8 (2.6)	296 (97.4)		44 (15.0)	249 (85.0)		3 (1.0)	300 (99.0)		7 (2.5)	275 (97.5)	
No	10 (4.8)	199 (95.2)		20 (9.7)	186 (90.3)		2 (0.9)	209 (99.1)		3 (1.5)	191 (98.5)	
Man has sex with man			0.585			0.419			0.283			0.637
Yes	18 (3.5)	498 (96.5)		65 (12.9)	437 (87.1)		6 (1.2)	511 (98.8)		10 (2.1)	471 (97.9)	
No	1 (4.2)	23 (95.8)		4 (16.0)	21 (84.0)		1 (4.0)	24 (96.0)		-	22 (100.0)	
Sexual partner in the last 12 months			0.972			0.054			0.488			0.298
0-1	4 (3.2)	122 (96.8)		11 (9.1)	110 (90.9)		1 (0.8)	125 (99.2)		3 (2.7)	110 (97.3)	
>1	4 (3.3)	129 (96.7)		21 (17.5)	99 (82.5)		2 (1.6)	120 (98.4)		1 (0.9)	116 (99.1)	
Condom			0.029			0.468			0.081			0.504
Yes	6 (7.3)	76 (92.7)		8 (10.3)	70 (89.7)		3 (3.7)	78 (96.3)		1 (1.3)	76 (98.7)	
No	10 (2.3)	422 (97.7)		56 (13.2)	367 (86.8)		4 (0.9)	431 (99.1)		9 (2.2)	393 (97.8)	

**Tabela 5:** Razão de prevalência (RP) bruta e ajustada e intervalo de confiança de 95% (IC95%) dos desfechos HIV, sífilis, hepatites B e C com as variáveis sociodemográficas e de vulnerabilidade. Complexo Penal Masculino de Tubarão e Florianópolis, 2023-2024 (n=555)

	HIV		Sífilis		Hepatite B		Hepatite C	
	RP bruta	RP ajustada	RP bruta	RP ajustada	RP bruta	RP ajustada	RP bruta	RP ajustada
Faixa etária (18-29 anos)	1,01	1,01	-	-	1,01	1,01	1,01	1,01
	(1,01; 1,03)	(0,99; 1,02)			(1,00; 1,02)	(1,01; 1,02)	(1,00; 1,03)	(1,01; 1,02)
Raça/cor da pele branca	0,99	0,99	1,02	1	-	-		
	(0,97; 1,01)	(0,96; 1,01)	(0,99; 1,05)	(0,96; 1,04)				
Cirurgia prévia	0,99	0,99	-	-	0,99	0,99	0,99	0,99
	(0,97; 1,00)	(0,97; 1,00)			(0,98; 1,00)	(0,99; 1,00)	(0,98; 1,01)	(0,98; 1,01)
Transfusão de sangue	-	-	1,03	1,02	-	-	-	-
			(1,00; 1,06)	(0,99; 1,07)				
Compartilha objetos	-	-	0,97	1,01	-	-	-	-
			(0,93; 1,01)	(0,96; 1,06)				
Uso de droga injetável	-	-	-	-	0,97	0,97	-	-
					(0,94; 1,01)	(0,94; 1,01)		
Uso de droga inalatória	1,01	1,01	0,97	0,97	-	-	-	-
	(0,99; 1,03)	(0,99; 1,03)	(0,94; 1,00)	(0,93; 1,01)				
Parceiros sexuais nos últimos 12 meses	-	-	1,05	1,05	-	-	-	-
			(1,00; 1,11)	(0,99; 1,10)				
Uso de preservativo	0,98	0,99	-	-	0,99	0,99	-	-
	(0,95; 1,00)	(0,96; 1,02)			(0,97; 1,01)	(0,97; 1,01)		

When the variable was analyzed as continuous, the number of sexual partners in life and the past 12 months was associated with reactive serology for syphilis. Those who tested positive for syphilis had a median of 100 sexual partners in their lifetime, compared to a median of 30 among those who tested negative (p-value 0.005). The same was observed regarding the number of partners in the last 12 months. Individuals with reactive syphilis serology had a median of four partners in the last 12 months, compared to a median of one partner among those with non-reactive results (p-value 0.035).

## Discussion

This study found a prevalence of syphilis (12.4%), HIV (3.4%), and hepatitis B (1.3%) and C (1.8%) among incarcerated men. The sample consisted predominantly of young, White, low-educated, single individuals. The sociodemographic profile was consistent with findings in the literature, indicating social vulnerability associated with structural and behavioral factors among incarcerated individuals and with risk factors for high STI prevalence ^9^ ^,^ ^10^.

Among vulnerable groups, young people stood out as targets for specific intersectoral policies. Racial distribution is noteworthy because, although white people predominate in the sample, the prevalence of syphilis was higher among black/brown people. More attention should be paid to the social determinants that affect this group. Low educational attainment reinforces the need for health education and promotion strategies that address cognitive and social barriers. 

The rise in syphilis cases has raised concern among health authorities ^5^ ^,^ ^11^, and this trend was also observed in the incarcerated population in this study. Rapid tests performed on men in the prison complex in Salvador, with 6,180 inmates, were evaluated, and a prevalence of 7.5% of syphilis was found using rapid serological screening tests ^12^. In 12 prison centers in the Central-West region, the prevalence of active syphilis among incarcerated men was 9.4%, based on blood tests ^13^. Considering the non-incarcerated population, among men who have sex with men, the overall prevalence of syphilis was found to be 7.5%. However, rates of 10.6% were reported in Latin America and the Caribbean ^14^. The present study revealed syphilis as the STI with the highest prevalence rate.

Brazil has experienced an increase in acquired syphilis rates, with men representing the majority of cases and the South region having the highest detection rate ^15^. Syphilis is a treatable disease, but reinfection can occur when sexual partners are not treated, and condoms are not used ^16^. In this study, a statistically significant association was found between syphilis infection and a higher number of sexual partners. Although the number of sexual partners may not differ significantly from the general population, risky practices and irregular condom use need to be continuously addressed, particularly during health campaigns within the prison system. The predominantly heterosexual profile of the incarcerated population does not eliminate the need for policies targeting men who have sex with men, as the prison environment may inhibit the acknowledgment of diverse sexual practices.

A potential strategy under investigation is the use of DoxyPEP for individuals who have had two or more STI in recent months or multiple STI simultaneously. Nevertheless, doxycycline prophylaxis is only effective for chlamydia and syphilis, with variable effectiveness for gonorrhea. Thus, condom use and systematic testing remain essential ^17^. Syphilis retesting among incarcerated individuals is recommended for treatment monitoring, reinfection control, and identifying new infections acquired during incarceration. The lack of retesting data in medical records hindered the evaluation of this phenomenon.

HIV prevalence in this population is considered high but consistent with the literature. In Santa Cruz do Sul, Rio Grande do Sul, a prevalence of 4.9% was found among 349 incarcerated individuals, based on laboratory testing by the Central Public Health Laboratory ^6^. Globally, HIV infection rates among incarcerated populations range from 0.0% to 14.5% ^5^. In Salvador, the rate was 1.2% ^12^, and a recent systematic review found an average HIV rate of 3.4% among incarcerated men, similar to that found in this study ^18^.

Although HIV/AIDS detection rates in Brazil declined in 2022, the decrease was smaller among men, especially among younger men and men who have sex with men. An increase in reported cases was also observed among Black/Brown individuals and those with lower education levels. In 2022, Santa Catarina ranked third among Brazilian states in detection rates ^19^. Sexual activity among incarcerated men does occur, although the extent is unknown due to the stigma and taboo surrounding the issue within the prison population ^20^. Moreover, condoms are only available during conjugal visits and not within cells.

With appropriate antiretroviral treatment and the use of barrier methods such as condoms, sexually active lives without risk to HIV-negative partners are possible. Nonetheless, the stigma surrounding the disease persists in Brazilian society ^21^. Another preventive option not yet implemented in the prison system is pre-exposure prophylaxis (PrEP), which could be effective given the vulnerability of incarcerated individuals ^22^.

Infection rates for hepatitis B and C were relatively low compared to other studies of incarcerated populations ^2^ ^,^ ^4^ ^,^ ^5^ ^,^ ^6^ ^,^ ^18^ ^,^ ^20^ ^,^ ^23^. Infection with hepatitis B and C was positively associated with older age. Currently, surgeries and transfusions involving blood and blood products are very safe for HIV and other bloodborne STI, including viral hepatitis. However, older individuals may not have received hepatitis B immunoprophylaxis prior to exposure and may have been exposed to contaminated medical materials ^24^. 

Although hepatitis B vaccination is mandatory, complete vaccination coverage was considered low. It was not possible to determine whether vaccination occurred inside or outside the prison system. Low compliance with hepatitis B vaccination was also observed among prisoners in Turkey ^25^. 

The global prevalence of hepatitis C among incarcerated individuals is 15.1%, and chronic hepatitis B is 4.8% ^26^. Brazilian data on viral hepatitis indicate high infection rates for hepatitis B and C, with higher prevalence among older men with low educational levels ^27^. In a systematic review that estimated the global prevalence among incarcerated individuals, hepatitis C had an average rate of 17.7% and hepatitis B of 5.2% ^18^. In Latin America and the Caribbean, an analysis of 73 studies involving 230,000 incarcerated individuals found global prevalence rates of 7.0% for hepatitis C and 1.0% for hepatitis B ^28^.

Coinfection among the tested infections is explained by shared transmission routes (sexual, bloodborne), as well as vulnerability factors such as low immunity and open entry points, especially in ulcerative STI like syphilis. This is a population with high vulnerability to STI due to incarceration, sexual exposure, low education, limited knowledge of these conditions, drug use, sharing of items, and high-risk behaviors ^29^. Approximately one-third of participants reported a current or previous STI. It was not possible to determine infections acquired during incarceration due to poor retesting records for individuals who were not diagnosed upon entry.

Prisons and other closed environments are often overlooked in terms of healthcare and scientific research. Incarceration can serve as a key moment for diagnosis and treatment, aiming to break transmission chains, promote education for reintegration into society, and prevent new STI, thereby reducing public health costs. Public policies involving regular testing, combined prevention (such as pre-exposure prophylaxis), and health education are essential to address the high infection rates observed.

This study has limitations, and its findings should be interpreted with caution. Although probabilistic sampling was used, some participants refused to take part or were excluded due to incomplete questionnaires or missing medical record data. The participants’ level of understanding may have influenced their responses to self-administered questionnaires. Screening tests may produce false-positive or false-negative results. Among the positive cases, laboratory confirmation of the infection was not possible in the medical records for only two participants. Therefore, only the serological screening test was used. There was no record of post-treatment monitoring or systematic retesting of non-reactive individuals to detect new infections acquired in prison. Given the study design, causality could not be established; however, the identified associated factors may generate hypotheses for future research.

Although there are other studies on STI among incarcerated men in Brazil, no recent research has been conducted, especially in Santa Catarina. These findings can support the planning of effective prison-based interventions. This study has a good sample size, which makes it representative of the location studied. The use of rapid tests in all Brazilian prison units-even if mostly at prison entry-is a highly sensitive diagnostic method, and its results are important for monitoring STI in this population.

In conclusion, the prevalence of HIV was 3.4%; syphilis, 12.4%; hepatitis B, 1.3%; and hepatitis C, 1.8%. Syphilis was associated with a higher number of sexual partners, while hepatitis B and C were associated with older age.

## Data Availability

Research data is available upon request.

## References

[1] World Prison Brief, Institute for Crime & Justice Policy Research (2022). Highest to lowest - Prison population total.

[2] Defante Ferreto LE, Guedes S, Braz Pauli F, Soligo Rovani S, Aní Caovilla Follador F, Paula Vieira A (2021). Seroprevalence and associated factors of HIV and Hepatitis C in Brazilian high-security prisons: A state-wide epidemiological study. PLoS One.

[3] Lima SS (2019). O cuidado aos usuários de drogas em situação de privação de liberdade. Physis.

[4] Krieguer D, da Silva LR, Johnson AJ, Wang EC (2019). Sexually transmitted infections detected during and after incarceration among people with human immunodeficiency virus: prevalence and implications for screening and prevention. Sex Transm Dis.

[5] Seyedalinigah S, Asadollahi-Amin A, Yousefi F, Janfeshan K, Ramezani M, Mehrtak M (2022). Prevalence of sexually transmitted infections and associated risk behaviors in prisoners: a systematic review. Health Sci Rep.

[6] Machado F, Souza L, Oliveira T, Dias E, Moura A (2019). Seroprevalence of HIV, hepatitis B e C e syphilis infection in prisoners of the central region of Rio Grande do Sul, Brazil. Mundo Saude.

[7] Dolan K, Wirtz AL, Moazen B, Ndeffo-Mbah M, Galvani A, Kinner SA (2016). Global burden of HIV, viral hepatitis, and tuberculosis in prisoners and detainees. Lancet.

[8] Saita NM, Gonçalves MD, Picanço MO, Dias LS, Bastos C, Quintana MS (2021). Determinants of coinfection tuberculosis and HIV in prisons in Brazil. Infect Dev Ctries.

[9] Sánchez A, Nogueira V, Garcia C, Pacheco J, Silveira R (2021). Mortalidade e causas de óbitos nas prisões do Rio de Janeiro, Brasil. Cad Saude Publica.

[10] Malvasi PA, Dantas HS, Manzalli SF (2022). Direitos humanos e saúde: reflexões sobre vida e política no contexto da população carcerária. Saude Soc.

[11] Ramchandani MS, Cannon CA, Marra CM (2023). Syphilis: A modern resurgence. Infect Dis Clin North Am.

[12] Leite AGDS, Silva ACS, Souza LG, Ramos VR (2022). Rapid tests for HIV, syphilis, and chronic hepatitis in a prison population in a prison complex in Salvador (BA), Brazil. Cien Saude Colet.

[13] Correa ME, Figueiró-Filho EA, Freitas AD, Cabral EA, Junior LB, Santos AD (2017). High prevalence of Treponema pallidum infection in Brazilian prisoners. Am J Trop Med Hyg.

[14] Tsuboi M, Evans J, Davies EP, Rowley J, Korenromp EL, Clayton T (2021). Prevalence of syphilis among men who have sex with men: a global systematic review and meta-analysis from 2000-20. Lancet Glob Health.

[15] Brasil, Ministério da Saúde, Secretaria de Vigilância em Saúde (2022). Boletim Epidemiológico: Sífilis, 2022.

[16] Luo Z, Chen Q, Wang L, Zhang L, Zhang Y, Liu L (2017). Factors associated with syphilis treatment failure and reinfection: a longitudinal cohort study in Shenzhen, China. BMC Infect Dis.

[17] Luetkemeyer AF, Donnell D, Dombrowski JC, Amico KR, Buchbinder S, Kirkcaldy RD (2023). Postexposure doxycycline to prevent bacterial sexually transmitted infections. N Engl J Med.

[18] Favril L, Rich JD, Hard J, Fazel S (2024). Mental and physical health morbidity among people in prisons: an umbrella review. Lancet Public Health.

[19] Brasil, Ministério da Saúde, Secretaria de Vigilância em Saúde e Meio Ambiente (2023). Boletim Epidemiológico: HIV/AIDS, 2023.

[20] Melendez-Torres G, Bourne A, Hickson F, Reid D, Weatherburn P (2022). eHealth interventions to address HIV and other sexually transmitted infections, sexual risk behavior, substance use, and mental ill-health in men who have sex with men: systematic review and meta-analysis. JMIR Public Health Surveill.

[21] de Andrade SLE, Moreira Freire ME, Collet N, Brandão GCG, Souza MHDN, Nogueira JA (2022). Structure of social networks of people living with HIV and AIDS. Rev Esc Enferm USP. Rev Esc Enferm USP.

[22] Jones MD, Barnes JN, Wilson LM, Freeman AE (2023). Examining the awareness, acceptability, and adoption of conventional and non-conventional forms of pre-exposure prophylaxis (PrEP) for HIV prevention among jail-involved Black sexual minority men (BSMM) and Black transgender women (BTW) in two diverse US cities. AIDS Behav.

[23] Puga MAM, Ferreira LL, Santos AS, Braga JU, Bastos FI (2019). Screening for HBV, HCV, HIV and syphilis infections among bacteriologically confirmed tuberculosis prisoners: an urgent action required. PLoS One.

[24] Guntipalli P, Pandit V, Rao VL, Nadella RK (2021). Worldwide prevalence, genotype distribution and management of hepatitis C. Acta Gastroenterol Belg.

[25] Kose S, Nazlican E, Tatar B, Yildirim B, Caglayan FN, Koksal I (2019). Hepatitis B and hepatitis C in prisons: a prevalence study. Int J Prison Health.

[26] UNAIDS (2019). HIV e pessoas em prisões e outros ambientes fechados. Série de fichas informativas sobre Direitos Humanos.

[27] Brasil, Ministério da Saúde, Secretaria de Vigilância em Saúde e Meio Ambiente (2023). Boletim Epidemiológico: Hepatites Virais, 2023.

[28] Magri MC, Manchiero C, Dantas BP, Bernardo WM, Abdala E, Tengan FM (2025). HBV, HCV and HIV among inmates in Latin America and the Caribbean: A systematic review and meta-analysis. Trop Med Int Health.

[29] do Nascimento CT, Pena DZ, Giuffrida R, Bandeira Monteiro FN, da Silva FA, Flores EF (2020). Prevalence and epidemiological characteristics of inmates diagnosed with infectious diseases living in a region with a high number of prisons in São Paulo state, Brazil. BMJ Open.

