# Supplementary material for: Prevalence of sexually transmitted infections among incarcerated men in Prison Complex: a cross-sectional study, Santa Catarina, 2023-2024
Source: Epidemiol Serv Saude. 2026 Feb 16;35:e20250140. doi: 10.1590/S2237-96222026v35e20250140.en (PMC12923203; doi:10.1590/S2237-96222026v35e20250140.en)
Supplement: Translation

# Translation: Prevalência de infecções sexualmente transmissíveis entre homens privados de liberdade do complexo penal: estudo transversal, Santa Catarina, 2023-2024

**DOI:** 10.1590/S2237-96222026v35e20250140.pt

**Authors:** Aline Ferreira Farias, Maria Laura Gava, Symonara Karina Medeiros Faustino Vieira, Gabriela Sousa De Araujo, Cassandra Loureiro Mangabeira, Eliane Traebert, Jefferson Traebert, Daisson José Trevisol, Fabiana Schuelter-Trevisol

**Affiliations:** 1 Universidade do Sul de Santa Catarina, Programa de Pós-Graduação em Ciências da Saúde, Tubarão, SC, Brasil

**Keywords:** HIV, Sífilis, Hepatite B, Hepatite C, Prisioneiros.

## Abstract

**Objetivo:**

Estimar a prevalência e os fatores associados de HIV, hepatites virais B e C e sífilis em homens privados de liberdade do Complexo Penal de Santa Catarina.

**Métodos:**

Tratou-se de estudo transversal com homens privados de liberdade em três unidades prisionais em Santa Catarina de agosto de 2023 a janeiro de 2024. Foram aplicados questionários e revisão da ficha de saúde do participante, a partir de amostragem aleatória simples. Foram calculadas as razões de prevalência brutas e ajustadas e os intervalos de confiança entre os desfechos e as variáveis independentes.

**Resultados:**

Foram estudados 555 homens. A média de idade foi 31,8±9,5 anos. O perfil sociodemográfico predominante foi de jovens, brancos, com baixa escolaridade e solteiros. Na amostra, foram identificados comportamentos de risco como tatuagem (87,2%), uso de drogas inalatórias (55,5%) e baixa adesão ao uso de preservativo (15,2%). A prevalência geral das infecções sexualmente transmissíveis (IST) testadas foi 16,8%, e 32,6% autorrelataram IST atual ou pregressa. A prevalência para HIV foi 3,4%; sífilis, 12,4%; hepatite B, 1,3%; e hepatite C, 1,8%. A sífilis se associou ao maior número de parceiros sexuais, e as hepatites B e C, à idade mais avançada.

**Conclusão:**

As taxas de prevalência de IST foram mais altas que na população em geral a partir do diagnóstico na entrada do sistema prisional. Houve falhas no acompanhamento de adesão ao tratamento e retestagem a fim de interromper a cadeia de transmissão.

**Aspectos éticos**

Esta pesquisa respeitou os princípios éticos, obtendo os seguintes dados de aprovação:

Comitê de ética em pesquisa: Universidade do Sul de Santa Catarina

Número do parecer: 5.591.802

Data de aprovação: 19/8/2022

Certificado de apresentação de apreciação ética: 59773922.2.0000.5369

Comitê de ética em pesquisa: Universidade do Sul de Santa Catarina

Número do parecer: 6.087.745

Data de aprovação: 29/5/2023

Certificado de apresentação de apreciação ética: 68717023.8.0000.0261

## Introdução

A população carcerária brasileira é a terceira maior do mundo ^1^. O contexto do encarceramento no Brasil é marcado por condições adversas, como superlotação, precariedade estrutural, falta de higiene, violência, deficiências na assistência à saúde e relações de poder desiguais ^2^ ^,^ ^3^. Tais fatores contribuem para um ambiente altamente vulnerável, o que favorece comportamentos de risco que aumentam a transmissão de agentes infecciosos. Nesse cenário, as infecções sexualmente transmissíveis (IST), como os vírus da imunodeficiência humana (HIV), sífilis, hepatite B e hepatite C, apresentam prevalência significativamente mais alta entre pessoas privadas de liberdade do que na população geral ^4^ ^,^ ^5^.

Essas infecções são associadas a práticas como relações sexuais sem preservativo, uso de drogas e compartilhamento de objetos cortantes ou de higiene pessoal ^4^ ^,^ ^6^. O alto índice de IST nessa população representa um desafio significativo para a saúde pública, especialmente considerando que as pessoas privadas de liberdade integram o grupo das populações-chave - aquelas mais expostas à infecção e com acesso limitado à prevenção e ao tratamento ^7^ ^,^ ^8^. Em 2017, 3,8% das pessoas privadas de liberdade no mundo viviam com HIV e 15,1% com hepatite C ^7^.

No sistema prisional de Dallas, Texas, índices de IST em ambientes carcerários permaneceram alarmantes, o que reforçou a necessidade de monitoramento e políticas públicas eficazes ^4^. Além dos impactos individuais, os altos índices de infecção representaram risco coletivo dentro e fora das prisões. Nas unidades prisionais do Rio de Janeiro, as mortes por doenças infecciosas foram cinco vezes mais frequentes do que na população geral, sendo o HIV/aids responsável por 43,0% desses óbitos ^9^.

As prisões configuram ambientes de alta complexidade, onde fatores como condições socioeconômicas desfavoráveis, acesso limitado a serviços de saúde e comportamentos de risco, como o compartilhamento de seringas e as relações sexuais desprotegidas entre homens, favorecem a disseminação de IST ^8^ ^,^ ^10^. 

Este estudo teve como objetivo estimar a prevalência e os fatores associados à infecção por HIV, hepatites B e C e sífilis em homens privados de liberdade no complexo penal de Santa Catarina.

## Métodos

Delineamento do estudo

Tratou-se de estudo transversal. 

Contexto

Foram estudados os homens privados de liberdade da Penitenciária e do Presídio Masculino de Florianópolis e da Penitenciária Masculina de Tubarão em Santa Catarina entre agosto de 2023 e janeiro de 2024. A Penitenciária de Florianópolis possuía, à época, 1.384 vagas (na ocasião da pesquisa, estava com 1.561 homens privados de liberdade); o Presídio, 475 vagas; e a Penitenciária de Tubarão, 370 vagas. O número de pessoas por cela é bastante variável, mas em média possui de seis a oito homens privados de liberdade por cela.

Participantes

Foram incluídos todos os homens privados de liberdade que realizaram os testes rápidos para HIV, sífilis e hepatites virais B e C e que concordaram em participar do estudo a partir de convite individual, a fim de garantir sua autonomia, com a assinatura do registro de consentimento livre e esclarecido. 

Foram excluídos os que não informaram nome ou matrícula, o que impossibilitava a coleta dos dados relativos aos testes rápidos e confirmatórios e às condições de saúde. Também foram excluídos aqueles internados no Hospital de Custódia e Tratamento Psiquiátrico da Penitenciária de Florianópolis.

Variáveis

As variáveis de interesse ao estudo incluíram dados sociodemográficos e antecedentes de saúde e comportamentos de risco, conforme a seguir. 

Dados sociodemográficos: idade categorizada em faixas etárias (18-29, 30-39, 40-49, 50-59, ≥60 anos) raça/cor da pele (branca, preta/parda, indígena, amarela), escolaridade (ensino fundamental incompleto e completo, ensino médio incompleto e completo, ensino superior incompleto e completo) e estado civil (solteiro, casado/amasiado, separado/divorciado, viúvo). 

Antecedents de saúde e comportamentos de risco: histórico de cirurgia (sim, não), transfusão de sangue prévia (sim, não), realização de tatuagem (sim, não) e *piercing* (sim, não), uso de drogas ilícitas inalatórias (sim, não) e injetáveis (sim, não), compartilhamento de objetos como lâminas de barbear (sim, não), se fez/faz sexo no sistema prisional (sim, não), orientação sexual (heterossexual, homossexual, bissexual), número de parceiros sexuais na vida (dicotomizado pela mediana em 0 a 34, >34) e nos últimos 12 meses (0 a 1 e >1), uso de preservativo (sempre, na maioria das vezes, eventualmente, nunca), vacinação contra hepatite B (sim, não) e diagnóstico prévio autorreferido de IST (sim, não). 

As variáveis dependentes do estudo foram a infecção por HIV (reagente, não reagente), sífilis (reagente, não reagente), hepatite B (reagente, não reagente) e hepatite C (reagente, não reagente).

Fontes de dados/mensuração

As variáveis de interesse foram coletadas com instrumento criado pelos pesquisadores e autoaplicável aos participantes, com questões fechadas e de múltipla escolha. Informações adicionais foram coletadas dos prontuários médicos do setor saúde e transcritas para o banco de dados. Foram verificados os resultados dos testes rápidos e confirmatórios para HIV, sífilis e hepatites B e C na entrada do sistema prisional. Nas informações obtidas diretamente com os participantes e sem possibilidade de checagem de dados, no caso de não saber a informação ou recusa em responder, a categoria de resposta foi padronizada como dado ignorado.

Viés

O instrumento de coleta de dados não foi validado. Embora tenha sido feita amostragem probabilística, algumas recusas e alguns impedimentos pela alta periculosidade dos participantes puderam influenciar os resultados.

Tamanho do estudo

O cálculo da amostra foi realizado pelo programa OpenEpi e teve como população total 2.260 indivíduos, prevalência do desfecho desconhecida (P=50%), limite de confiança de 95% resultando em amostra mínima de 329 participantes, sendo adicionado mais 30,0% como margem de segurança para eventuais perdas. Foi realizada amostragem aleatória simples, a partir da numeração dos pavilhões, e conforme a possibilidade de coleta, pois, em alguns setores, não foi permitida a entrada por motivos de segurança ou ausência de local adequado para a aplicação dos questionários.

Coleta de dados

Os participantes sorteados foram convidados a participar do estudo e, mediante aceite e anuência do registro de consentimento livre e esclarecido, foram submetidos a um questionário autoaplicável, na presença de um dos pesquisadores a fim de sanar dúvidas quanto ao instrumento de coleta de dados.

O questionário incluiu a coleta de dados sociodemográficos relativos a encarceramento, vida sexual e comportamentos de risco. Os questionamentos feitos se basearam em instrumentos utilizados em estudos anteriores com essa temática, com base na revisão de literatura. Utilizaram-se as salas de aula dos setores autorizados para a coleta de dados. Pequenos grupos de participantes foram encaminhados, a partir do sorteio das celas, até se atingir o tamanho amostral. Posteriormente, revisaram-se os prontuários médicos, ao quais se encontravam no Sistema Integrado de Segurança Pública de Santa Catarina, para averiguação dos resultados dos testes sorológicos para HIV, sífilis e hepatites B e C e variáveis clínicas complementares. 

Os testes rápidos empregados eram os disponibilizados pela Secretaria de Estado da Saúde de Santa Catarina e apresentaram alta sensibilidade e especificidade. Como os testes foram feitos na entrada do sistema prisional, e alguns participantes estavam encarcerados havia alguns anos, as marcas dos testes podem ter tido variação, e não havia essa especificação nos registros. 

Os testes rápidos foram exames de imunocromatografia para detectação de anticorpos anti-HIV, detectação de anticorpos IgG e IgM para sífilis, anti-hepatite C e o HBsAg. Todos os resultados reagentes foram encaminhados para o serviço de atendimento especializado do município para os testes confirmatórios, segundo o protocolo do Ministério da Saúde, e a notificação do caso. A sífilis foi confirmada pelo Veneral Disease Research Laboratory. Nos testes positivos para HIV, foi realizado segundo exame sorológico e confirmado pela carga viral por reação em cadeia da polimerase (PCR), assim como foi empregada a detecção por PCR para hepatites B e C. Embora nos prontuários houvesse o registro de infecção confirmada, nem sempre os testes confirmatórios feitos foram arquivados no Complexo Prisional. 

A retestagem de controle dos marcadores sorológicos para sífilis deve ser feita a cada três meses para o controle de tratamento e para detectar casos de reinfecção. Observou-se que nem sempre esse procedimento foi seguido, bem como o reteste dos casos não reagentes durante o tempo de permanência na instituição. Por esse motivo, para este estudo, foi considerado o teste positivo na entrada do sistema prisional. 

Métodos estatísticos

As informações obtidas foram anexadas e organizadas em um banco de dados no Microsoft Excel 2016. A análise estatística foi realizada no software SPSS v. 21 (IBM, Armonk, Nova York, Estados Unidos). Utilizou-se o teste de Kolmogorov-Smirnov para avaliar a normalidade dos dados quantitativos. 

As variáveis quantitativas foram descritas por meio de medidas de tendência central e dispersão dos dados. As variáveis contínuas como idade e número de parceiros na vida e nos últimos 12 meses foram dicotomizadas pela mediana da distribuição. As variáveis qualitativas foram descritas por meio de frequência absoluta e percentual. As diferenças nas proporções foram testadas pelo teste de qui-quadrado de Pearson e diferenças de médias pelo teste *t* de Student, ou equivalentes não paramétricos, conforme a adequação dos dados. O nível de significância estatística adotado foi 5% com intervalo de confiança de 95%.

A análise estatística foi conduzida no programa Statistical Package for Social Sciences (SPSS) versão 18.0 (SPSS Inc., Chicago, Illinois, Estados Unidos. As variáveis quantitativas foram descritas por meio de medidas de tendência central e dispersão. Razões de prevalência (RP) brutas e ajustadas e seus intervalos de confiança de 95% (IC95%) foram estimados por meio de regressão de Poisson com estimador robusto. Para o ajuste das variáveis de confundimento nos três modelos estudados, foram incluídas as variáveis com p-valor<0,20 na análise bivariada. O nível de significância adotado foi 5%.

## Resultados

Foram estudados 555 homens privados de liberdade entre agosto de 2023 a janeiro de 2024. A média de idade dos participantes foi 31,8 anos (desvio-padrão ± 9,5), com idade mínima de 18 anos e máxima de 76 anos (Tabela 1). Observaram-se os dados relacionados a fatores de risco e de vulnerabilidade associados às IST de acordo com informações autorrelatadas pelos participantes do estudo (Tabela 2). Apresentaram-se os dados de fatores de risco comportamentais relativos a comportamento sexual, autorrelatadas pelos participantes do estudo, assim como declaração de ciência sobre contaminação de IST atual ou pregressa e vacinação para hepatite B (Tabela 3).

De acordo com os testes rápidos realizados na entrada do sistema prisional atual, 93 (16,8%) apresentaram resultado reagente para, pelo menos, uma das quatro infecções testadas. A maior prevalência foi sífilis (69; 12,4%); em seguida, HIV (19; 3,4%) e hepatites virais C (10; 1,8%) e B (7; 1,3%). Houve dez casos de coinfecção, três casos de HIV/sífilis, dois casos de HIV/hepatite B, dois de HIV/hepatite B/hepatite C, dois de sífilis/hepatite C e um de sífilis/hepatite B. 

Apresentaram-se os resultados da análise bivariada entre as variáveis independentes e os quatro desfechos do estudo (Tabela 4). A idade mais avançada e a não adesão ao uso de preservativos foram fatores associados à infecção pelo HIV. Na hepatite B, a idade mais avançada e o uso de drogas injetáveis também se associaram à infecção. Nos casos de sífilis e hepatite C, nenhuma variável independente se associou ao desfecho. Contudo, ao se realizar a análise bruta e ajustada das variáveis independentes aos desfechos, apenas a idade mais avançada se associou de forma independente à infecção pela hepatite B (Tabela 5).

**Tabela 1:** Perfil sociodemográfico dos homens privados de liberdade. Complexo Penal Masculino de Tubarão e Florianópolis, 2022-2023 (n=555)

Variável	n (%)
Faixa etária (anos)	
18-29	272 (49,0)
30-39	178 (32,1)
40-49	75 (13,5)
50-59	22 (4,0)
≥60	8 (1,4)
Raça/cor da pele	
Branca	277 (49,9)
Preta/parda	268 (48,3)
Indígena	7 (1,3)
Amarela	3 (0,5)
Escolaridade	
Ensino fundamental incompleto	285 (51,4)
Ensino fundamental completo	74 (13,3)
Ensino médio incompleto	116 (20,9)
Ensino médio completo	48 (8,6)
Ensino superior incompleto	22 (4,0)
Ensino superior completo	10 (1,8)
Estado civil	
Solteiro	303 (54,6)
Casado/amasiado	213 (38,4)
Separado/divorciado	33 (5,9)
Viúvo	6 (1,1)

**Tabela 2:** Fatores de riscos e vulnerabilidade associados às infecções sexualmente transmissíveis dos homens privados de liberdade. Complexo Penal Masculino de Tubarão e Florianópolis, 2023-2024 (n=555)

Variável	n (%)
Cirurgia pregressa	
Sim	270 (48,7)
Não	271 (48,8)
Ignorado	14 (2,5)
Recebeu transfusão sanguínea	
Sim	159 (28,7)
Não	367 (66,1)
Ignorado	29 (5,2)
Possui tatuagem	
Sim	490 (88,2)
Não	63 (11,4)
Ignorado	2 (0,4)
Usa *piercing*	
Sim	44 (8,0)
Não	503 (90,6)
Ignorado	8 (1,4)
Usuário de droga injetável	
Sim	48 (8,6)
Não	466 (84,0)
Ignorado	41 (7,4)
Usuário de droga inalatória	
Sim	308 (55,5)
Não	218 (39,3)
Ignorado	29 (5,2)
Compartilha objetos de higiene pessoal	
Sim	107 (19,3)
Não	407 (73,3)
Ignorado	41 (7,4)

**Tabela 3:** Dados relacionados a questões e comportamentos sexuais, incluindo tratamento e prevenção às infecções sexualmente transmissíveis dos homens privados de liberdade. Complexo Penal Masculino de Tubarão e Florianópolis, 2023-2024 (n=555)

Variável	n (%)
Orientação sexual	
Heterossexual	530 (95,5)
Homossexual	5 (0,9)
Bissexual	20 (3,6)
Fez/faz sexo dentro do estabelecimento prisional	
Sim	143 (25,8)
Não	401 (72,3)
Ignorado	11 (1,9)
Número de parceiros sexuais na vida	
0-34	128 (23,1)
>34	125 (22,5)
Ignorado	302 (54,4)
Número de parceiros últimos 12 meses	
0-1	267 (48,1)
>1	95 (17,1)
Ignorado	193 (34,8)
Tem ou teve infecções sexualmente transmissíveis	
Sim	178 (32,6)
Não	352 (64,5)
Ignorado	25 (2,9)
Uso de preservativo	
Sempre	83 (15,0)
Na maioria das vezes	114 (20,5)
Eventualmente	188 (33,8)
Nunca	143 (25,8)
Ignorado	27 (4,9)
Foi vacinado contra hepatite B	
Sim	122 (22,0)
Não	135 (24,3)
Ignorado	298 (53,7)

**Tabela 4:** Associação entre HIV, sífilis, hepatites B e C e variáveis de interesse. Complexo Penal Masculino de Florianópolis e Tubarão, 2023-2024 (n=555)

.	HIV			Sífilis			Hepatite B			Hepatite C		
	Reagente	Não	p-valor	Reagente	Não reagente	p-valor	Reagente	Não	p-valor	Reagente	Não reagente	p-valor
	n (%)	reagente		n (%)	n (%)		n (%)	reagente		n (%)	n (%)	
		n (%)						n (%)				
Faixa etária (anos)			0,042			0,307			0,008			0,052
18-29	5 (1,9)	261 (98,1)		38 (14,6)	222 (85,4)		-	268 (100,0)		2 (0,8)	250 (99,2)	
>29	14 (5,1)	260 (94,9)		31 (11,6)	236 (88,4)		7 (2,6)	267 (97,4)		8 (3,2)	243 (96,8)	
Raça/cor da pele			0,092			0,182			0,488			0,354
Branca	13 (4,9)	254 (95,1)		29 (11,1)	232 (88,9)		4 (1,5)	264 (98,5)		6 (2,4)	241 (97,6)	
Não branca	6 (2,2)	267 (97,8)		40 (15,0)	226 (85,0)		3 (1,1)	271 (98,9)		4 (1,6)	252 (98,4)	
Escolaridade (anos)			0,374			0,402			0,228			0,23
0-8	14 (4,0)	332 (96,0)		48 (14,0)	295 (86,0)		6 (1,7)	345 (98,3)		8 (2,5)	312 (97,5)	
>8	5 (2,6)	189 (97,4)		21 (11,4)	163 (88,6)		1 (0,5)	190 (99,5)		2 (1,1)	181 (98,9)	
Cirurgia prévia			0,111			0,449			0,062			0,196
Sim	13 (4,9)	253 (95,1)		36 (14,0)	222 (86,0)		6 (2,3)	259 (97,7)		7 (2,8)	246 (97,2)	
Não	6 (2,3)	255 (97,7)		30 (11,7)	226 (88,3)		1 (0,4)	263 (99,6)		3 (1,3)	235 (98,7)	
Transfusão de sangue			0,633			0,073			0,35			0,457
Sim	6 (3,9)	148 (96,1)		13 (8,7)	137 (91,3)		3 (1,9)	151 (98,1)		2 (1,4)	142 (98,6)	
Não	11 (3,1)	347 (96,9)		51 (14,5)	301 (85,5)		4 (1,1)	357 (98,9)		7 (2,1)	326 (97,9)	
Tatuagem			0,623			0,271			0,443			0,683
Sim	17 (3,6)	460 (96,4)		58 (12,4)	411 (87,6)		7 (1,5)	475 (98,5)		9 (2,0)	436 (98,0)	
Não	2 (3,2)	60 (96,8)		10 (17,5)	47 (82,5)		-	59 (100,0)		1 (1,8)	56 (98,2)	
Piercing			0,55			0,863			0,546			0,542
Sim	1 (2,4)	41 (97,6)		6 (14,0)	37 (86,0)		-	44 (100,0)		1 (2,7)	36 (97,3)	
Não	18 (3,7)	474 (96,3)		62 (13,0)	414 (87,0)		7 (1,4)	483 (98,6)		9 (2,0)	451 (98,0)	
Compartilhamento			0,313			0,078			0,715			0,394
Sim	2 (1,9)	103 (98,1)		19 (17,9)	87 (82,1)		1 (0,9)	105 (99,1)		1 (1,0)	99 (99,0)	
Não	14 (3,5)	382 (96,5)		44 (11,5)	340 (88,5)		4 (1,0)	392 (99,0)		8 (2,2)	360 (97,8)	
Uso droga injetável			0,487			0,547			0,013			0,577
Sim	2 (4,3)	45 (95,7)		7 (15,6)	38 (84,4)		3 (6,3))	45 (93,8)		1 (2,4)	41 (97,6)	
Não	15 (3,3)	438 (96,7)		55 (12,4)	388 (87,6)		3 (0,7)	451 (99,3)		8 (1,9)	415 (98,1)	
Droga inalatória			0,193			0,081			0,665			0,362
Sim	8 (2,6)	296 (97,4)		44 (15,0)	249 (85,0)		3 (1,0)	300 (99,0)		7 (2,5)	275 (97,5)	
Não	10 (4,8)	199 (95,2)		20 (9,7)	186 (90,3)		2 (0,9)	209 (99,1)		3 (1,5)	191 (98,5)	
Homem faz sexo com homem			0,585			0,419			0,283			0,637
Sim	18 (3,5)	498 (96,5)		65 (12,9)	437 (87,1)		6 (1,2)	511 (98,8)		10 (2,1)	471 (97,9)	
Não	1 (4,2)	23 (95,8)		4 (16,0)	21 (84,0)		1 (4,0)	24 (96,0)		-	22 (100,0)	
Parceiros sexuais em 12 meses			0,972			0,054			0,488			0,298
0-1	4 (3,2)	122 (96,8)		11 (9,1)	110 (90,9)		1 (0,8)	125 (99,2)		3 (2,7)	110 (97,3)	
>1	4 (3,3)	129 (96,7)		21 (17,5)	99 (82,5)		2 (1,6)	120 (98,4)		1 (0,9)	116 (99,1)	
Preservativo			0,029			0,468			0,081			0,504
Sim	6 (7,3)	76 (92,7)		8 (10,3)	70 (89,7)		3 (3,7)	78 (96,3)		1 (1,3)	76 (98,7)	
Não	10 (2,3)	422 (97,7)		56 (13,2)	367 (86,8)		4 (0,9)	431 (99,1)		9 (2,2)	393 (97,8)	

**Tabela 5:** Razão de prevalência (RP) bruta e ajustada e intervalo de confiança de 95% (IC95%) dos desfechos HIV, sífilis, hepatites B e C com as variáveis sociodemográficas e de vulnerabilidade. Complexo Penal Masculino de Tubarão e Florianópolis, 2023-2024 (n=555)

	HIV		Sífilis		Hepatite B		Hepatite C	
	RP bruta	RP ajustada	RP bruta	RP ajustada	RP bruta	RP ajustada	RP bruta	RP ajustada
Faixa etária (18-29 anos)	1,01	1,01	-	-	1,01	1,01	1,01	1,01
	(1,01; 1,03)	(0,99; 1,02)			(1,00; 1,02)	(1,01; 1,02)	(1,00; 1,03)	(1,01; 1,02)
Raça/cor da pele branca	0,99	0,99	1,02	1	-	-		
	(0,97; 1,01)	(0,96; 1,01)	(0,99; 1,05)	(0,96; 1,04)				
Cirurgia prévia	0,99	0,99	-	-	0,99	0,99	0,99	0,99
	(0,97; 1,00)	(0,97; 1,00)			(0,98; 1,00)	(0,99; 1,00)	(0,98; 1,01)	(0,98; 1,01)
Transfusão de sangue	-	-	1,03	1,02	-	-	-	-
			(1,00; 1,06)	(0,99; 1,07)				
Compartilha objetos	-	-	0,97	1,01	-	-	-	-
			(0,93; 1,01)	(0,96; 1,06)				
Uso de droga injetável	-	-	-	-	0,97	0,97	-	-
					(0,94; 1,01)	(0,94; 1,01)		
Uso de droga inalatória	1,01	1,01	0,97	0,97	-	-	-	-
	(0,99; 1,03)	(0,99; 1,03)	(0,94; 1,00)	(0,93; 1,01)				
Parceiros sexuais nos últimos 12 meses	-	-	1,05	1,05	-	-	-	-
			(1,00; 1,11)	(0,99; 1,10)				
Uso de preservativo	0,98	0,99	-	-	0,99	0,99	-	-
	(0,95; 1,00)	(0,96; 1,02)			(0,97; 1,01)	(0,97; 1,01)		

Quando analisada a variável como contínua, número de parceiros na vida e nos últimos 12 meses se associou à sorologia reagente para sífilis. Aqueles que apresentaram resultado sorológico reagente para sífilis tiveram mediana de 100 parcerias sexuais na vida em comparação à mediana de 30 naqueles com resultado sorológico não reagente (p-valor 0,005). Isso também foi evidenciado quanto ao número de parcerias nos últimos 12 meses. Aqueles que apresentaram resultado sorológico reagente para sífilis tiveram mediana de quatro parcerias sexuais no último ano em comparação à mediana de um naqueles com resultado sorológico não reagente (p-valor 0,035).

## Discussão

Neste estudo, verificou-se a prevalência de sífilis (12,4%), HIV (3,4%) e hepatites virais B (1,3%) e C (1,8%) entre homens privados de liberdade. A amostra foi composta por essa população com predomínio de jovens, da raça/cor de pele branca, com baixa escolaridade e solteiros. O perfil sociodemográfico foi concordante com os achados da literatura, o que indicou a vulnerabilidade social, associada a fatores estruturais e comportamentais das pessoas encarceradas e os fatores de risco para a alta prevalência de IST ^9^ ^,^ ^10^.

Entre os grupos vulneráveis, destacou-se a população jovem que é alvo de políticas intersetoriais específicas. A distribuição racial merece atenção, pois, embora haja predominância da raça/cor branca na amostra, a prevalência de sífilis foi maior entre pessoas negras/pardas, exigindo mais atenção para os determinantes sociais que afetam esse grupo. A baixa escolaridade reforça a necessidade de estratégias de educação e promoção da saúde que considerem as barreiras cognitivas e sociais. 

O aumento dos casos de sífilis tem alertado as autoridades de saúde ^5^ ^,^ ^11^, e observou-se esse aumento também na população carcerária como evidenciado neste estudo. Foram avaliados os testes rápidos feitos em homens do complexo prisional de Salvador, com 6.180 pessoas privadas de liberdade, e foi encontrada a prevalência de 7,5% de sífilis a partir do uso de testes rápidos de triagem sorológica ^12^. Em 12 centros prisionais da região Centro-Oeste, encontrou-se a taxa de 9,4% de sífilis ativa entre os homens encarcerados, a partir de exames sanguíneos ^13^. Considerando a população não encarcerada, entre homens que fazem sexo com homens, foi encontrada a prevalência global de sífilis de 7,5%, contudo taxas 10,6% foram evidenciadas na América Latina e Caribe ^14^. Os dados deste estudo revelaram que a sífilis foi a IST com maior taxa de prevalência.

O Brasil apresentou aumento das taxas de sífilis adquirida, sendo os homens a maior parcela desses casos e a região Sul com a maior taxa de detecção ^15^. A sífilis é uma doença tratável, mas casos de reinfecção podem ocorrer quando não há o tratamento de parceiros sexuais e o não uso de preservativo ^16^. Neste estudo, houve associação estatisticamente significativa entre a infecção pela sífilis e o maior número de parcerias sexuais. Ainda que o número de parceiros sexuais não difira significativamente da população geral, as práticas de risco e o uso irregular de preservativos precisam ser abordados de forma contínua, especialmente durante as campanhas de saúde no sistema prisional. O perfil majoritariamente heterossexual da população carcerária não elimina a necessidade de políticas voltadas para homens que fazem sexo com homens, pois o ambiente prisional pode inibir o reconhecimento de práticas sexuais diversas.

Uma alternativa que tem sido estudada é o uso da DoxiPEP para pessoas que tiveram duas ou mais IST nos últimos meses ou várias IST simultaneamente. Contudo, a profilaxia com doxiciclina só é eficaz para clamídia e sífilis, tendo eficácia variável para o gonococo. Assim, o uso de preservativos e a testagem sistemática são sempre indicados ^17^. Sugere-se a retestagem de sífilis entre pessoas privadas de liberdade tanto para o controle de tratamento e reinfecção quanto para os casos novos adquiridos dentro do sistema prisional. A escassez de dados de retestagem nos prontuários médicos dos participantes impediu a avaliação desse fenômeno.

A prevalência de HIV nessa população é considerada elevada, mas concordante com a literatura. Em Santa Cruz do Sul, no Rio Grande do Sul, entre 349 pessoas privadas de liberdade, encontrou-se a soroprevalência para HIV de 4,9%, a partir das análises sanguíneas do Laboratório Central de Saúde Pública ^6^. Entre pessoas privadas de liberdade no mundo, a taxa de infecção pelo HIV variou de 0,0% a 14,5% ^5^. Entre homens privados de liberdade de Salvador, a taxa encontrada foi 1,2% ^12^, e, em recente revisão sistemática, a taxa média de HIV entre homens encarcerados foi 3,4%, muito similar a este estudo ^18^.

Embora no Brasil tenha havia queda na taxa de detecção de HIV/aids em 2022, essa desaceleração foi menor no sexo masculino, especialmente nos homens que fazem sexo com homens e naqueles mais jovens. Também foi evidenciado aumento dos casos notificados entre pessoas da raça/cor da pele preta/parda e com menor escolaridade. Em, 2022, Santa Catarina apareceu como o terceiro estado no ranque da taxa de detecção ^19^. A relação sexual entre os homens privados de liberdade ocorre, embora essa taxa seja desconhecida por ser considerado um assunto tabu e com preconceito pela própria população carcerária ^20^. Ademais, a disponibilidade de preservativo ocorre quando há visita íntima, e não nas celas.

Por meio do tratamento antirretroviral adequado e do uso de métodos de barreira como o preservativo, pode-se garantir vida sexual ativa, sem risco para o parceiro soronegativo. Contudo, o preconceito que envolve a doença ainda é presente na sociedade brasileira ^21^. Outra opção preventiva, mas que ainda não é utilizada no sistema prisional, é a profilaxia pré-exposição que poderia ser uma tecnologia eficaz para evitar a infecção pelo HIV considerando a vulnerabilidade das pessoas privadas de liberdade ^22^.

As taxas de infecção de hepatites B e C foram relativamente baixas em comparação a outros estudos com pessoas privadas de liberdade ^2^ ^,^ ^4^ ^,^ ^5^ ^,^ ^6^ ^,^ ^18^ ^,^ ^20^ ^,^ ^23^. A infecção por hepatites B e C se associaram positivamente à idade mais avançada. Atualmente, as cirurgias e transfusões de sangue e hemoderivados são práticas assistenciais muito seguras tanto para a infecção pelo HIV como para hepatites virais e outras IST transmitidas por via sanguínea. Todavia, pessoas mais velhas podem não ter recebido a imunoprofilaxia contra hepatite B antes da exposição viral e terem sido expostas a materiais médico hospitalares contaminados ^24^. 

Apesar de a vacinação para hepatite B ser obrigatória, a prevalência do esquema vacinal completo foi considerada baixa. Não foi possível determinar se a vacinação foi realizada dentro ou fora dos estabelecimentos prisionais. Baixa adesão à vacinação para hepatite B também foi evidenciada em prisioneiros da Turquia ^25^. 

A prevalência mundial de hepatite C entre pessoas encarceradas é de 15,1% e de hepatite B crônica de 4,8% ^26^. Dados brasileiros sobre as hepatites virais revelaram grande percentual de infecção pelas hepatites B e C, sendo mais predominantes em homens, com idade mais avançada e baixa escolaridade ^27^. Em revisão sistemática que estimou a prevalência global entre pessoas privadas de liberdade, a hepatite C teve taxa média de 17,7% e a hepatite B de 5,2% ^18^. Na América Latina e Caribe, análise de 73 estudos, com 230 mil pessoas privadas de liberdade, encontrou prevalência global de hepatite C de 7,0% e de hepatite B de 1,0% ^28^.

A coinfecção entre as infecções testadas é explicada tanto pela via comum de transmissão (sexual, sanguínea) como pela vulnerabilidade devido à baixa imunidade e porta de entrada no caso de IST ulcerosa como a sífilis. Essa é uma população com grande vulnerabilidade para IST devido a encarceramento, exposição sexual, baixo nível de escolaridade e pouco conhecimento sobre esses agravos, além do uso de drogas, compartilhamentos de objetos e promiscuidade ^29^. Cabe ressaltar que, aproximadamente, um terço dos participantes afirmou ter IST atual ou pregressa. Não foi possível determinar infecções que tenham acontecido durante o encarceramento, pois havia fragilidade dos registros de retestagem entre os homens privados de liberdade que não tiveram a infecção diagnosticada na entrada do complexo penal.

As prisões e outros ambientes fechados são por vezes negligenciados quanto à assistência à saúde e para a condução de estudos científicos sobre essa realidade. Durante o encarceramento, torna-se um momento oportuno de diagnóstico e tratamento, com vistas a interromper a cadeia de transmissão, educar para a reintegração na sociedade e prevenir novas IST, com redução de custos para a sociedade. Políticas públicas que incluam testagem regular, prevenção combinada (como a profilaxia pré-exposição) e educação em saúde são fundamentais para enfrentar os altos índices de infecção observados.

Este estudo apresentou limitações, por isso os dados devem ser interpretados com cautela. Embora tenha sido feita uma amostra probabilística, houve recusa de participação entre alguns homens privados de liberdade e algumas exclusões por falha de preenchimento do instrumento de coleta ou registros dos prontuários médicos. O questionário autoaplicável pode sofrer influência do grau de compreensão dos participantes. Testes de triagem podem apresentar resultados falso positivos e falso negativos. Entre os positivos, em apenas dois participantes não foi possível a confirmação laboratorial da infecção registrada no prontuário, sendo usado apenas o teste de triagem sorológica. Não foi feito o registro do controle pós-tratamento, tampouco o registro de retestagem sistemática daqueles com resultados não reagentes a fim de detectar novas infecções adquiridas no sistema prisional. Pelo delineamento do estudo, também não foi possível determinar causalidade, embora os fatores associados possam levantar hipóteses para estudos futuros.

Embora haja outros estudos sobre IST com homens privados de liberdade no Brasil, não há pesquisas recentes, especialmente em Santa Catarina. Os dados podem contribuir para o planejamento de intervenções efetivas no contexto prisional. Este estudo tem bom tamanho amostral, o que se torna representativo do local estudado. O emprego de testes rápidos em todas as unidades prisionais brasileiras, ainda que majoritariamente na entrada do sistema prisional, é um método diagnóstico com alta sensibilidade, cujos resultados são importantes no monitoramento das IST nessa população.

Conclui-se que a prevalência de HIV foi 3,4%; sífilis, 12,4%; hepatite B, 1,3%; e hepatite C, 1,8%. A sífilis se associou ao maior número de parceiros sexuais, e as hepatites B e C, à idade mais avançada.

## Data Availability

Os dados de pesquisa só estão disponíveis mediante solicitação.
